# Efficacy and Safety of Microneedling Combined With Mesotherapy for Androgenic Alopecia: A Retrospective Study Comparing Four Treatment Protocols

**DOI:** 10.1002/hsr2.72760

**Published:** 2026-07-01

**Authors:** Elham Behrangi, Roya Zeinali, Paria Jafari, Zahra Nosrati, Azadeh Goodarzi

**Affiliations:** ^1^ Department of Dermatology, Hazrat Fatemeh Hospital, School of Medicine Iran University of Medical Sciences (IUMS) Tehran Iran; ^2^ School of medicine Iran University of Medical Sciences Tehran Iran

**Keywords:** androgenic alopecia, female pattern hair loss, finasteride, hair loss, latanoprost, male pattern hair loss, mesotherapy, microneedling

## Abstract

**Background and Aims:**

Androgenic alopecia (AGA) is a prevalent form of hair loss characterized by progressive miniaturization of hair follicles, significantly impacting patients’ self‐confidence and quality of life. Current FDA‐approved treatments demonstrate limited to moderate efficacy. Microneedling combined with mesotherapy techniques has emerged as a promising drug delivery system for enhancing therapeutic outcomes in hair loss disorders. This retrospective interventional study aimed to evaluate the efficacy and safety of four distinct microneedling‐based mesotherapy protocols for the treatment of androgenic alopecia.

**Methods:**

Sixty‐three patients with clinically confirmed AGA were allocated to four treatment groups. All groups received microneedling therapy combined with different mesotherapy formulations: Group I (*n* = 16) received 2 mL hyaluronic acid (HA); Group II (*n* = 20) received 1.5 mL HA plus 0.5 mL finasteride; Group III (*n* = 15) received 1.5 mL HA plus 0.5 mL latanoprost; and Group IV (*n* = 12) received 1 mL HA plus 0.5 mL finasteride and 0.5 mL latanoprost. Patients underwent six treatment sessions at 3–4 week intervals. Standardized digital photographs were obtained at baseline, 3 months, and 6 months. Efficacy was assessed using physician satisfaction scores (treating and blinded evaluators) and patient satisfaction scores. Safety was monitored after each treatment session.

**Results:**

Thirty‐nine women and 24 men completed the study. Women demonstrated significantly higher improvement rates (48%) compared to men (29%). Female pattern hair loss showed better response (45%) than male pattern AGA (37.5%). Participants under 30 years achieved higher improvement rates (47%) compared to those aged 30–49 years (42.9%). Group IV (combination therapy with finasteride and latanoprost) demonstrated the highest efficacy across both sexes and hair loss patterns. All treatment protocols were well‐tolerated with minimal adverse effects.

**Conclusions:**

The findings of the current study support the use of combination mesotherapy protocols for enhanced therapeutic outcomes in AGA management.

## Summary

- Combination mesotherapy (finasteride plus latanoprost) with microneedling demonstrates superior efficacy compared to monotherapy approaches in AGA treatment.
- Female patients, particularly those under 30 years with female pattern hair loss, show the highest response rates to microneedling‐based mesotherapy.
- The treatment protocol is well‐tolerated with minimal adverse effects, making it a viable option for patients seeking non‐surgical hair restoration.
- Standardized treatment intervals (3–4 weeks) over six sessions provide optimal outcomes while maintaining patient compliance.

## 1 Introduction

Androgenic alopecia (AGA), commonly known as male or female pattern hair loss, is a prevalent form of hair loss characterized by progressive miniaturization of hair follicles [1]. While this condition is primarily driven by androgens such as dihydrotestosterone (DHT), its etiology is considered multifactorial, involving genetic predisposition, hormonal influences, and environmental factors [2]. Although AGA typically begins in the third or fourth decade of life, hair loss can start shortly after puberty and gradually progress [3].

The classification system for AGA differs between male‐ and female‐pattern hair loss (MPHL and FPHL). In MPHL, the Norwood–Hamilton scale is used to evaluate the extent of hair loss, ranging from minimal recession (Type I) to severe hair loss (Type VI), with particular attention to frontotemporal recession and crown thinning. In FPHL, the Ludwig classification is employed, with Grade I indicating partial thinning at the crown, Grade II representing significant thinning, and Grade III denoting complete baldness at the crown [4, 5].

The prevalence of AGA varies significantly across different populations and age groups, with a general trend of increasing incidence with age. In Singapore, a higher prevalence was noted among Indians (87%) compared to Chinese (61%) [6]. In contrast, Caucasians generally report higher prevalence rates than Asians [7]. Recent Chinese college freshmen studies indicate that the prevalence of alopecia is increasing, affecting approximately 70% of men and 40% of women [8]. AGA often diminishes self‐confidence and negatively impacts quality of life [9].

Current treatment options for AGA remain limited. The FDA has approved only two medications for AGA: topical minoxidil (for both men and women) and oral finasteride (for men only). While these treatments can slow hair loss and promote regrowth in some patients, their efficacy is moderate, with response rates varying between 30% and 60%, and they require continuous use to maintain benefits [10]. Additionally, systemic side effects, particularly with finasteride, limit its acceptability among some patients.

Microneedling has emerged as a promising adjunctive treatment for AGA. The proposed mechanisms include: activation of stem cells in the hair follicle bulge area through mechanical stimulation; release of growth factors such as platelet‐derived growth factor (PDGF) and vascular endothelial growth factor (VEGF); enhanced dermal absorption of topically applied medications through creation of microchannels; and induction of dermal papilla neovascularization [11, 12].

Mesotherapy has also gained attention in recent years for its potential in treating various cosmetic concerns, especially hair loss [13]. Medications such as finasteride, minoxidil, latanoprost, hyaluronic acid (HA), and amino acids are administered in AGA [14]. Minoxidil, a vasodilator, promotes hair growth by lengthening the anagen phase, reducing the telogen phase, and enlarging miniaturized follicles. Finasteride inhibits the enzyme 5‐alpha reductase type 2, reducing the conversion of testosterone to DHT [15]. Latanoprost stimulates the anagen phase in telogen follicles by acting on prostaglandin F2α receptors in the dermal papilla and outer root sheath [16]. Despite the availability of various mesotherapy cocktails containing ingredients like hyaluronic acid and biotin, their efficacy remains insufficiently established in the literature.

Despite the theoretical advantages of combining microneedling with mesotherapy, there is limited clinical evidence comparing different mesotherapy formulations in this context. This study aimed to evaluate and compare the efficacy and safety of four distinct microneedling‐based mesotherapy protocols for AGA treatment, with specific attention to response variations based on patient demographics and hair loss patterns.

## 2 Materials and Methods

This retrospective interventional study included men and women aged 18 to 60, all with a confirmed diagnosis of androgenetic alopecia (AGA) by a reputable dermatologist from Rasool Akram Hospital between September 2022 and July 2023. Exclusion criteria encompassed patients with scalp infections, inflammatory diseases, malignancies, pregnant or lactating women, and those with a history of allergic reactions to mesotherapy ingredients.

All participants provided written informed consent prior to the procedure, ensuring their full understanding of the treatment process.

Eligible participants were divided into four treatment groups, each receiving different types of mesotherapy administered via microneedling.

Patients were divided into four groups based on the specific mesotherapy formulations used:

- Group I: 2 cc of HA
- Group II: HA 1.5 cc + finasteride 0.5 cc
- Group III: HA 1.5 cc + Latanoprost eyedrop 0.5 cc
- Group IV: HA 1 cc + topical finasteride 0.5 cc + Latanoprost 0.5 cc

Microneedling (Dr. Pen A6 Ultima, China) with single‐use 12‐pin cartridges was performed on all patients without anesthesia. A few drops of mesotherapy medication were first applied to the scalp, followed by the application of a micro‐needling device with a pick‐and‐place movement, set to a needle depth of 1.5–2 mm. The procedure was repeated 2–3 times until pinpoint bleeding occurred.

Treatment sessions were conducted every 3–4 weeks for 6 months.

Demographic data, including age, gender, pre‐treatment hair loss severity (based on the Ludwig and Hamilton‐Norwood scales), hair loss patterns (MPHL or FPHL), presence of hormonal imbalances or disorders, prior use of anti‐hair loss medications, and sessions conducted in the 6 month period were meticulously recorded in a checklist.

Standardized digital photographs were taken at baseline, the third month, and the sixth month using a Canon 750D camera. Efficacy was assessed by both the treating therapist and an independent dermatologist using a three‐point scale:

1: Slight improvement (≤ 25%)
2: Moderate improvement (26%–75%)
3: Significant improvement (≥ 75%)

Additionally, a blind dermatologist evaluated the efficacy of the treatments based on the checklist variables.

Participants also performed a self‐assessment of their satisfaction using the following scale:

1: Low satisfaction (≤ 25%)
2: Moderate satisfaction (26%–75%)
3: High satisfaction (≥ 75%)

To evaluate safety, potential side effects such as pain, localized redness, bruising, swelling, tenderness, and itching were documented before and after each session.

Categorical variables, including sex, age group, pattern of hair loss, mesotherapy group, physician satisfaction scores, and patient satisfaction scores, were summarized using absolute frequencies and percentages. Comparisons between categorical variables were conducted using the chi‐square test, in accordance with standard statistical methodology for categorical data. Statistical analyses were performed using IBM SPSS Statistics software (version 26.0; IBM Corp., USA). All statistical tests were two‐sided, and the a priori level of statistical significance was set at *p* < 0.05.

## 3 Results

In this study, 63 patients diagnosed with androgenetic alopecia (AGA), consisting of 39 women (61.9%) and 24 men (38.1%), were included. The participants had a mean age of 35.34 years, ranging from 21 to 53. Male pattern hair loss (MPHL) and female pattern hair loss (FPHL) were observed in 50.7% and 49.3% of patients, respectively. Only 7 out of 39 (17.95%) women had polycystic ovary disease (PCOD), and most participants (72.05%) were hormonally normal. A detailed breakdown of the demographic data can be found in Table 1.

**Table 1:** Demographic data of study participants.

Demographic data	Categories	Frequency (percent)
Sex	Men	24(38.1%)
	Women	39(61.9%)
Age	< 30	17(27%)
	30–49	42(66.7%)
	50≤	4(6.2%)
Hair loss pattern	Male	32(50.7%)
	M1	0(0%)
	M2	8(12.7%)
	M3	18(28.6%)
	M4	5(7.9%)
	M5	1(1.6%)
	Female	31(49.2%)
	F1	1(1.6%)
	F2	20(31.7%)
	F3	10(15.8%)
Hormonal disorder	Non	55(87.3%)
	PCOD	7(11.1%)
	Hypothyroid	1(1.6%)
Anti‐hair loss medications	None	45(71.4%)
	spironolactone	9(14.3%)
	oral finasteride	7(11.3%)
	spironolactone + oral finasteride	2(3.2%)
Meso‐needling sessions number	Less than 6	55.6%
	6	33.3%
	More than 6	11.1%
Mesotherapy group	Group I	25.4%
	Group II	31.7%
	Group III	23.8%
	Group IV	19%

**Figure 1:**
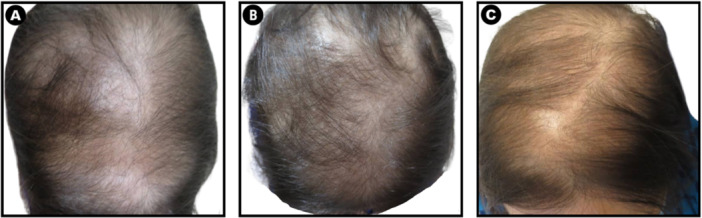
A 40‐year‐old woman with grade 3 female pattern androgenetic alopecia (AGA), without significant past medical history, was treated with spironolactone in group 4. Both the assessors and the patient reported significant satisfaction. The images show her condition before treatment (A), and after 3 months (B) and 6 months (C) of treatment.

**Figure 2:**
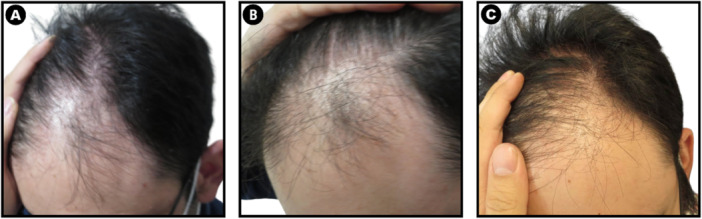
A 36‐year‐old man with grade 4 male pattern androgenetic alopecia (AGA) and no significant medical history or oral medication underwent more than six mesotherapy sessions in Group 2. Both the assessors and the patient reported significant satisfaction. The images show his condition before treatment (A), and after three months (B) and six months (C) of treatment.

The study also analyzed differences between various mesotherapy groups in terms of treatment outcomes, dermatologist assessments, and patient satisfaction. However, these differences were not statistically significant, as shown in Table 2 and Table 3. Figure 1 and Figure 2 illustrate a woman with FPHL and a man with MPHL during the study period.

**Table 2:** Treating and blind physician satisfaction scores across different groups according to three‐point scale. A *p*‐value < 0.05 is considered significant.

		Treating dermatology score					Blind dermatologist score				
		1	2	3	total	*p*‐value	1	2	3	total	*p* value
Frequency (number/percent)	Group I	8 (50%)	2 (12.5%)	6 (37.5%)	16	0.261	7 (43.8%)	3 (18.8%)	6 (37.5%)	16	0.507
	Group II	4 (20%)	6 (30%)	10 (50%)	20		4 (20.0%)	4 (20.0%)	12 (20.0%)	20	
	Group III	3 (20%)	5 (33.3%)	7 (46.7%)	15		6 (40.0%)	4 (26.7%)	5 (33.3%)	15	
	Group IV	3 (25%)	1 (8.3%)	8 (66.7%)	12		6 (50.0%)	3 (25.0%)	3 (25.0%)	12	
Total		18 (28.6%)	14 (22.2%)	31 (49.2%)	63		23 (36.5%)	14 (22.2%)	26 (41.3%)	63	

**Table 3:** Patient satisfaction scores in the studied groups according to three‐point scale. A *p*‐value < 0.05 is considered significant.

		Patient satisfaction score				
		1	2	3	Total	*p* value
Frequency (number/percent)	Group I	6/(37.5%)	6/(37.5%)	4/(25%)	16	0.923
	Group II	5/(25%)	7/(35%)	8/(40%)	20	
	Group III	5/(33.3%)	5/(33.3%)	5/(33.3%)	15	
	Group IV	3/(25%)	6/(50%)	3/(25%)	12	
Total		19/(30.2%)	24/(38.1%)	20/(31.7%)	63	

A comprehensive set of results, based on blind dermatologist assessment, is provided in the supplementary file. In summary, based on blind dermatologist assessment, treatment efficacy based on age was statistically significant (*p* = 0.023), with better outcomes observed in patients under 30 years old (Supplementary Figure S5). However, no significant differences were found concerning the type of mesotherapy material used (Supplementary Table S2). Regarding dermatologist satisfaction and the type of mesotherapy medication, gender (as shown in Supplementary Table S1) and hair loss pattern were statistically significant factors (*p* = 0.003 and *p* = 0.046, respectively) (Supplementary Table 4). Other variables were not statically significant (Supplementary Tables S3, S5–S7).

When comparing groups with statistically significant differences, we found that 48.77% of female patients achieved the best results, compared to 29.72% of male patients (Figure 2). Additionally, 45.2% of patients with FPHL achieved the best results, compared to 37.6% of those with MPHL (Supplementary Figure S11). Among those under 30 years old, 47.1% saw the best results, compared to 42.8% of those over 30 (Supplementary Figure S5). The combination of HA + Finasteride + Latanoprost had the most positive effect across both gender and hair loss pattern groups.

Importantly, no significant side effects were reported during the study period. The observed side effects were mild and temporary.

## 4 Discussion

There has been increasing attention toward alternative treatment methods for androgenic alopecia recently. Microneedling in combination with mesotherapy drugs has been investigated in several studies; however, the efficacy and safety of these drugs remain under debate.

This retrospective study aimed to determine the best mesotherapy combination in the mesoneedling technique and to assess the impact of gender, age, and pattern of hair loss on the response to mesotherapy. Although patient and physician satisfaction did not differ significantly between the various mesotherapy groups, these factors became relevant when age, sex, and pattern of hair loss were taken into account.

It is well established in the literature that combining microneedles with conventional AGA treatment, topical minoxidil 5%, significantly improves hair thickness compared to topical minoxidil alone [17, 18, 19]. Microneedling is an accessible and easy‐to‐use tool that yields satisfactory outcomes with minimal side effects, both through its regenerative effects and its ability to enhance drug delivery [11]. The stratum corneum layer is considered a barrier that reduces the efficacy of topical medications. With microneedling, medicines can pass this barrier, leading to higher efficacy [12]. Moreover, the injury and wound healing process induced by microneedling triggers the release of cytokines, platelet‐derived growth factors, activation of stem cells, increased expression of hair growth genes, vascular endothelial growth factor (VEGF), and proteins such as catenin B, Wnt3a, and Wnt10b [12]. In the present study, the majority of both patients and assessors reported improvement in all groups.

Although the mesotherapy technique for treating AGA has gained significant attention among dermatologists, the efficacy and safety have not been well studied [20]. Various mesotherapy medicines and cocktails are available on the market, though none have been approved by the FDA [14]. Mesotherapy utilizing vitamins and minerals alone was found to be more successful, patient‐acceptable, and tolerable compared to topical minoxidil 5% for female pattern hair loss (FPHL) [21]. Minoxidil mesotherapy for AGA has been evaluated in several studies with satisfactory results, showing superiority over topical application [20]. However, there is a lack of studies on other medications, which were explored in this study.

Finasteride, a 5‐alpha‐reductase inhibitor, reduces the conversion of testosterone to dihydrotestosterone (DHT), a major factor in hair follicle miniaturization. Topical application of finasteride has emerged as a promising option with a more favorable safety profile compared to oral administration [22]. Statistically significant improvements were reported in a review article with dutasteride mesotherapy, another 5‐alpha‐reductase inhibitor, with greater increases in hair shaft diameter observed when dutasteride was combined with biotin, pyridoxine, and d‐panthenol compared with dutasteride monotherapy [23]. The current study demonstrates that adding finasteride to the mesotherapy solution increased the patient satisfaction rate, and the response rate was higher in the combination group.

Latanoprost, a prostaglandin analog, has shown potential in treating androgenic alopecia (AGA) by enhancing hair growth through increasing hair follicle density [24]. In a 24‐week study involving men with mild AGA, daily application of latanoprost 0.1% increased hair density compared to baseline (*n* = 16, *p* < 0.001) and placebo‐treated sites (*p* = 0.0004) [16]. However, this study found that adding latanoprost to a mesotherapy solution did not result in a significant improvement in the satisfaction rate.

A case report by Melo et al. detailed that 20 bi‐weekly sessions of a sterile mesotherapy blend containing minoxidil, finasteride, biotin, and d‐panthenol, administered to a male patient with partial response to topical minoxidil and oral finasteride over 2 years, led to significant improvement in hair density and thickness [25]. In the current study, a mixture of mesotherapy drugs did not show significant improvement overall. However, there were notable differences based on age, gender, and pattern of hair loss, with the best response seen in men under 30. Additionally, the study duration and the number of intervention sessions were shorter than in similar studies, which may have influenced the results. More frequent sessions and shorter intervals could be recommended for men over 30 with male pattern hair loss (MPHL).

In 2019, Starace et al. conducted a study revealing differing response patterns to treatment between male and female pattern AGA [26]. Their findings indicated that the vertex area in male pattern AGA and the frontal scalp in female pattern AGA showed a more robust response to treatment. Similarly, our present study concluded that the vertex area demonstrated a higher response rate to microneedling compared to the bitemporal area.

According to the findings of this study, the response rate in women was much higher than in men. Specifically, 54% of women in Group 1 reported high satisfaction, compared to 0% of men, and 50% of FPHL patients and 25% of MPHL patients reported high satisfaction. This suggests that gender and pattern of hair loss significantly affect response rates. A study compared the efficacy of eight sessions of mesotherapy solution injections with daily topical application of 5% minoxidil over 4 months in men with AGA and found no significant improvement of mesotherapy over minoxidil [27]. However, another study assessed the efficacy of weekly mesotherapy in FPHL compared to daily application of 5% minoxidil. The mesotherapy group showed a greater improvement in the number of hair follicles after treatment (*p* = 0.007), indicating that it is a more effective, acceptable, and tolerable treatment compared to topical minoxidil for FPHL [28].

The most recent literature agrees that microneedling is well tolerated and associated with only mild and transient adverse events [17, 18, 19]. During the mesoneedling sessions in our study, two patients experienced erythema in the treated area, three reported pruritus, and three experienced temporary hair loss, which was attributed to the addition of latanoprost and resolved after discontinuation of this mesotherapy. Despite these minor issues, no major adverse effects were identified overall. This reinforces the safety profile of mesoneedling as a treatment for AGA.

Lastly, our study is distinctive for its analysis of demographic data, an aspect that has been underexplored in existing literature. By examining demographic factors, we broaden the understanding of variables that may influence treatment response and patient characteristics.

Several limitations of this study should be acknowledged. First, the retrospective, non‐randomized design limits causal inference and introduces potential selection bias. Additionally, the study relied on subjective satisfaction scores rather than objective measures such as hair count, density, or thickness measurements via trichoscopy or phototrichogram. Only 33.3% of patients completed all six planned sessions. This may reflect the incomplete collection of 6‐month outcome data for all participants, potentially introducing the risk that outcomes (satisfaction and improvement) are influenced by variation in treatment duration. Finally, the absence of a true control group (microneedling alone or no treatment) limits the ability to isolate the specific contribution of mesotherapy agents. Future research should address these limitations.

## 5 Conclusion

Microneedling combined with mesotherapy represents a safe and effective treatment option for androgenic alopecia, with particularly promising results in young women with female pattern hair loss. The combination protocol incorporating both finasteride and latanoprost showed the highest efficacy trend, suggesting potential synergistic benefits. While the treatment was well‐tolerated across all groups, larger randomized controlled trials with objective outcome measures are needed to confirm these findings and establish optimal treatment protocols. This study contributes valuable preliminary data supporting the use of combination microneedling‐mesotherapy approaches in AGA management and identifies key patient subgroups most likely to benefit from this intervention.

## Author Contributions

Contributions to the current study include E.B. in the study idea and design, literature review, and drafting and revising the manuscript critically for important intellectual content. Z.N. was involved in the proposal preparation, conducting the trial, data gathering, drafting the proposal, obtaining ethical committee approval, and revising the manuscript critically for important intellectual content. P.J. and R.Z. participated in the literature review, analysis, and interpretation of the revised version, and drafted the manuscript. A.G. supervised the study, gathered data, conducted literature reviews, drafted the manuscript, and served as the corresponding author. All authors have read and approved the final version to be published and agreed to be accountable for all aspects of the work.

## Funding

The authors have nothing to report.

## Ethics Statement

All information collected was kept confidential and evaluated without a specific name. This research was approved by the Research Council of Iran University of Medical Science under the ethics code number IR. IUMS. FMD. REC.1399.835.

## Conflicts of Interest

The authors declare no conflicts of interest.

## Supplementary Material

The supplementary file contains pie charts representing the data from the checklist, including gender (Supplementary Figure S1), age (Supplementary Figure S4), number of sessions (Supplementary Figure S7), pattern of hair loss (Supplementary Figure S10), grade of AGA (Supplementary Figure S13), PMH (Supplementary Figure S16), and oral medication (Supplementary Figure S19).

Additionally, blind dermatologist assessment scores based on these variables are shown in general (Supplementary Figures S2, S5, S8, S11, S14, S17, S20) and illustrated for four groups (Supplementary Tables S1–7 and Figures S3, S6, S9, S12, S15, S18, S21).

## Transparency Statement

The lead author, Dr. Elham Behrangi affirms that this manuscript is an honest, accurate, and transparent account of the study being reported; that no important aspects of the study have been omitted; and that any discrepancies from the study as planned (and, if relevant, registered) have been explained.

## Supporting information

Supporting File

## Data Availability

The data that support the findings of this study are available from the corresponding author upon reasonable request.
