# Supplementary material for: Efficacy and Safety of Microneedling Combined With Mesotherapy for Androgenic Alopecia: A Retrospective Study Comparing Four Treatment Protocols
Source: Health Sci Rep. 2026 Jul 1;9(7):e72760. doi: 10.1002/hsr2.72760 (PMC13323846; doi:10.1002/hsr2.72760)
Supplement: Supplementary file 1 — Supporting File [file HSR2-9-e72760-s001.docx]

**Supplementary File**

1. **Gender**:

This study comprises of 39 women (61.9%) and 24 men (38.1%). (Figure 1’)


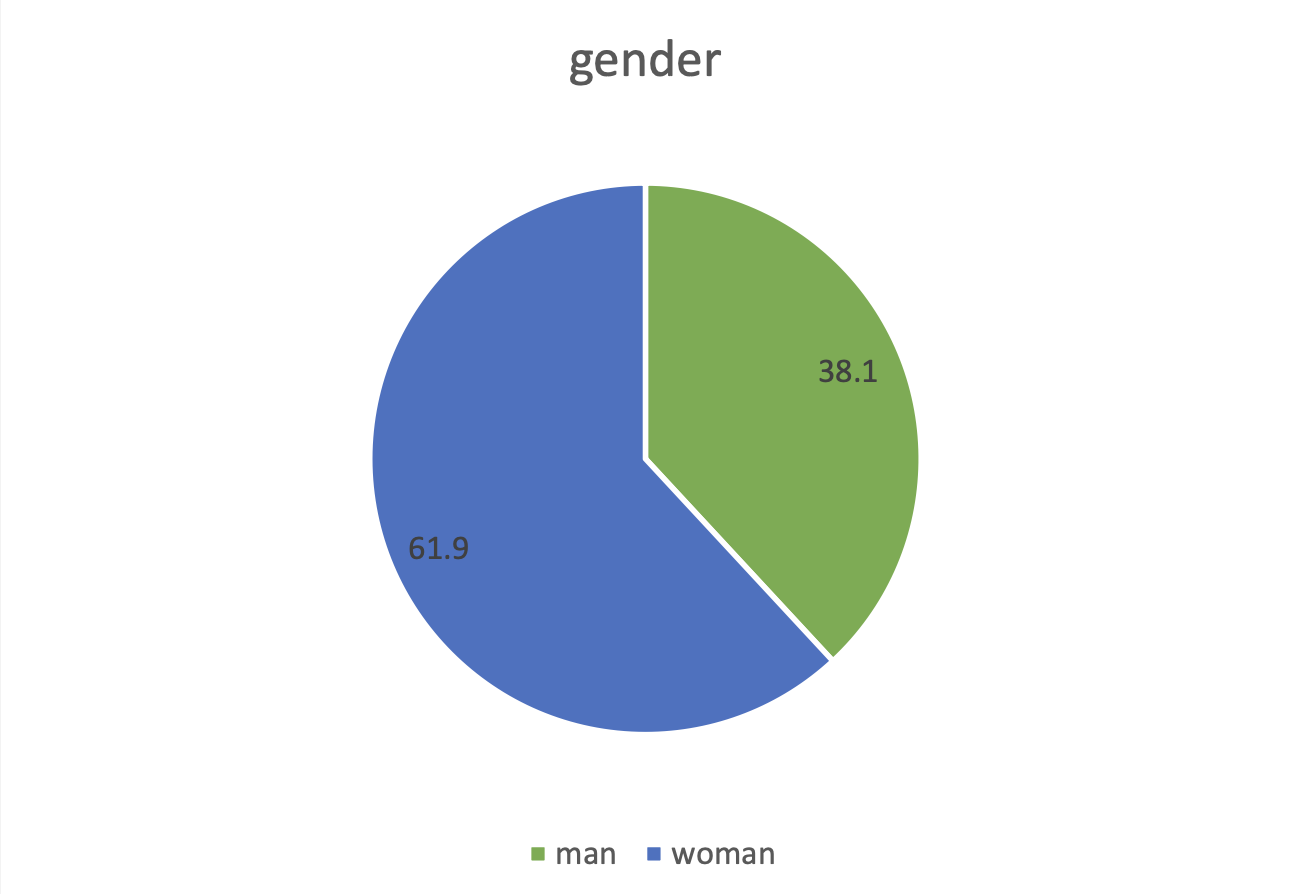


Figure 1': Distribution of Gender in the Current Study.

It was found that 48.77% of women (19 out of 39) achieved the highest level of satisfaction, whereas in the group of men, this figure was 29.72% (7 out of 24). This suggests that women benefit more from mesoneedling for AGA compared to men.


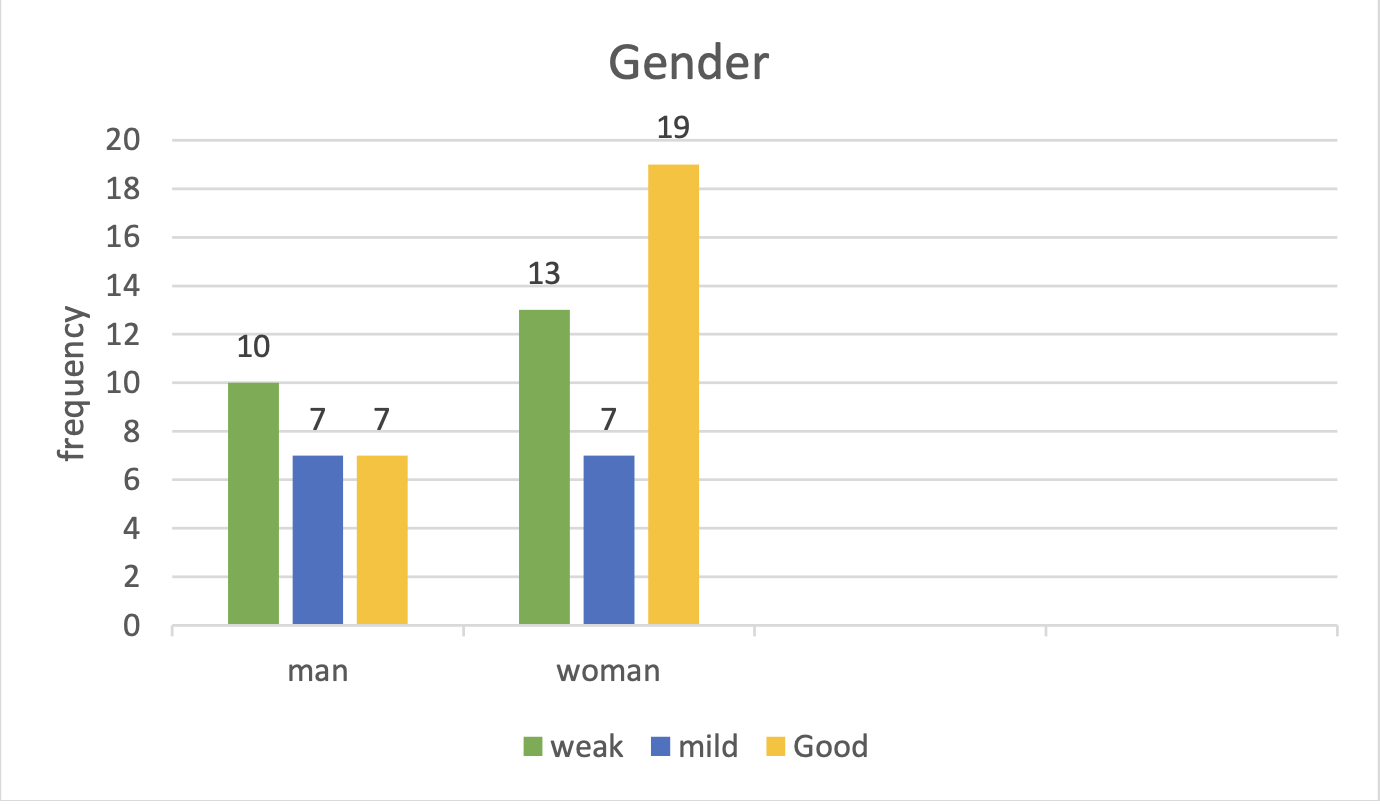


Figure 2': Assessment scores of blind dermatologists based on gender..

Using the chi-square test, the satisfaction of blind dermatologists based on gender was examined and found to be statistically non-significant (p-value = 0.286). However, the satisfaction score of blind dermatologists was found to be significant based on the mesotherapy group, when considering the sex variable (p-value = 0.003) (Table 1’) (Figure 3’).

Table 1': Assessment of Blind Dermatologists Based on Gender in Different Groups. A p-value < 0.05 is considered significant.

Figure 3': Frequency of Blind Dermatologist Assessments Based on Gender in Different Groups.

1. **Age**:

The mean age of patients at the start of the study was 35.34 years, with an age range of 21 to 53 years (Figure 4’).


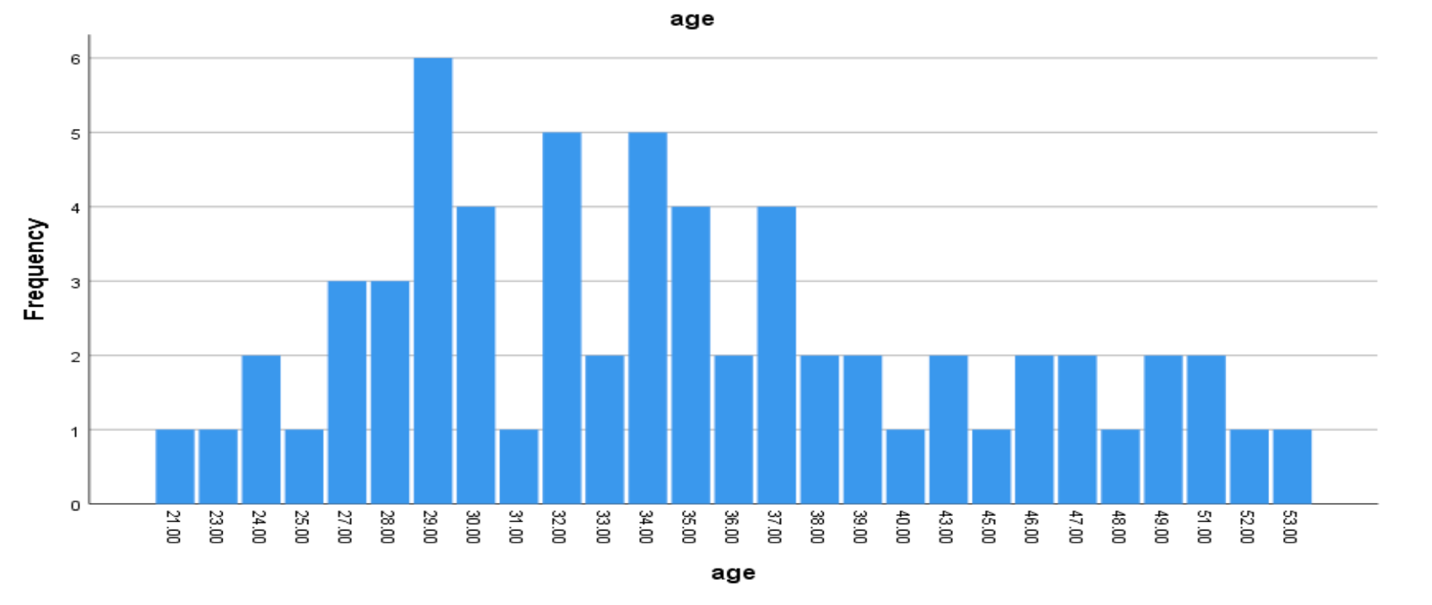


Figure 4': Age Range of Participants in This Study.

We found that 47.1% of individuals in the age group under 30 years old achieved the highest level of satisfaction. In contrast, 81% of individuals in the age group over 31 years old achieved the highest satisfaction level. This indicates that patients in the under-30 age group had higher levels of satisfaction compared to the older age groups (Figure 5’).


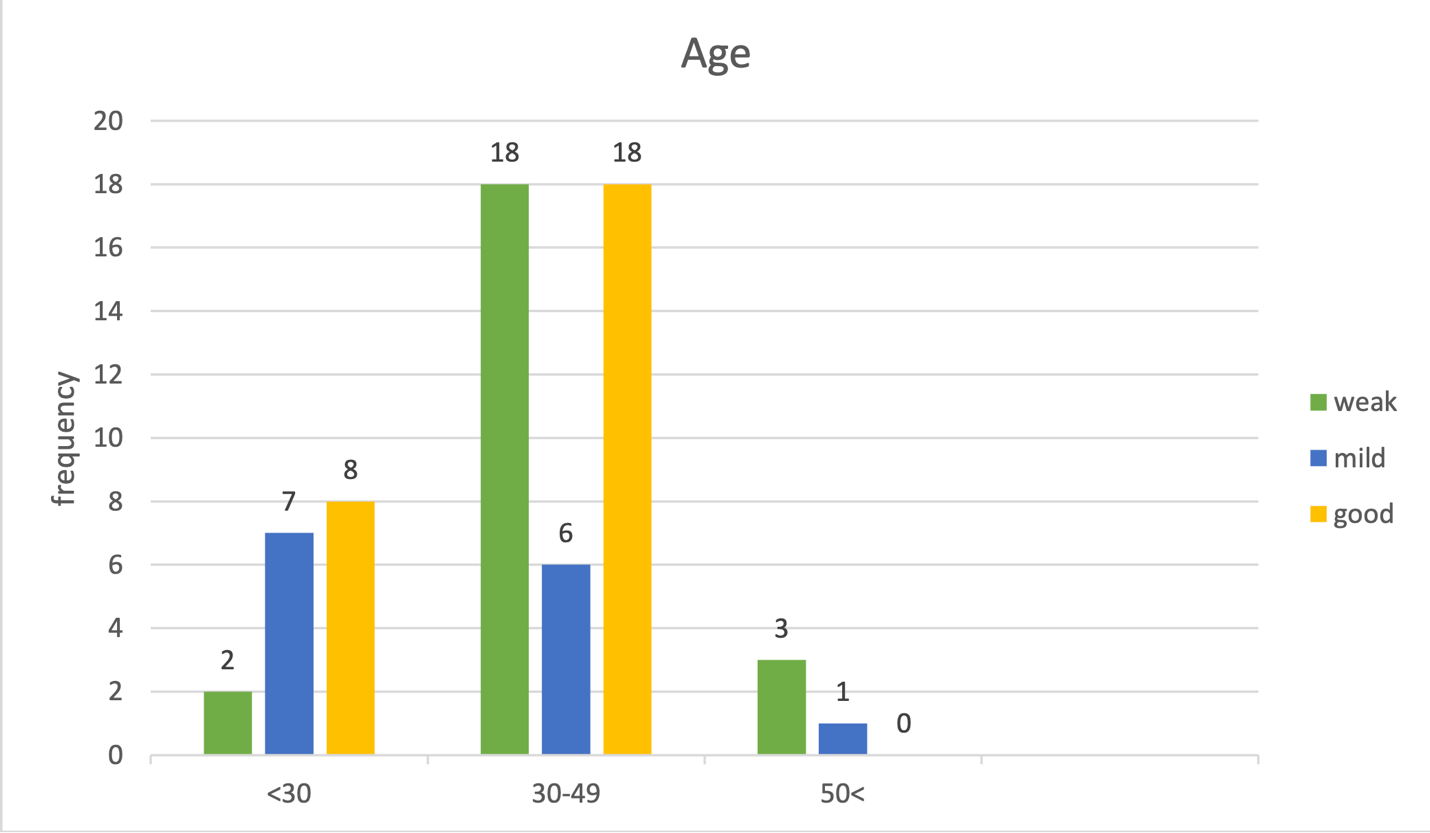


Figure 5': Frequency of Blind Dermatologist Assessments Based on Age.

.

There is a significant correlation between age groups and the satisfaction rate, based on blind dermatologist assessments (p-value = 0.023). Based on blind dermatologist evaluation, the satisfaction rate is not significantly different among groups based on age (p-value=0.135) (Table 2’) (Figure 6’).

Table 2': Blind Dermatologist Assessments Based on Age in Different Groups. A p-value < 0.05 is considered significant.

*Figure 6’: Frequency of Blind Dermatologist Assessments Based on Age in Different Groups.*

1. **Number of Sessions:**

Figure 7’ demonstrates the frequency of number of sessions in the study.


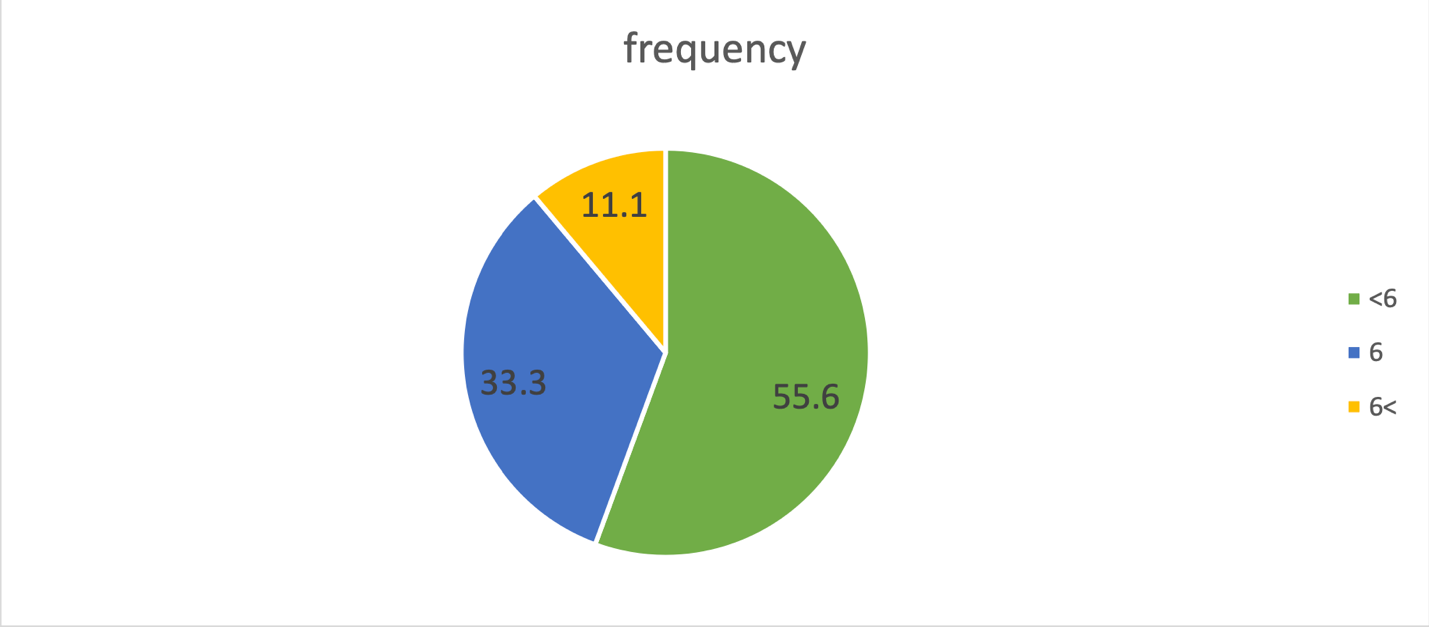


*Figure 7’: Frequency of Mesotherapy Sessions in This Study.*

Figure 8’ demonstrates the frequency of blond dermatologist assessment scores based on number of sessions.


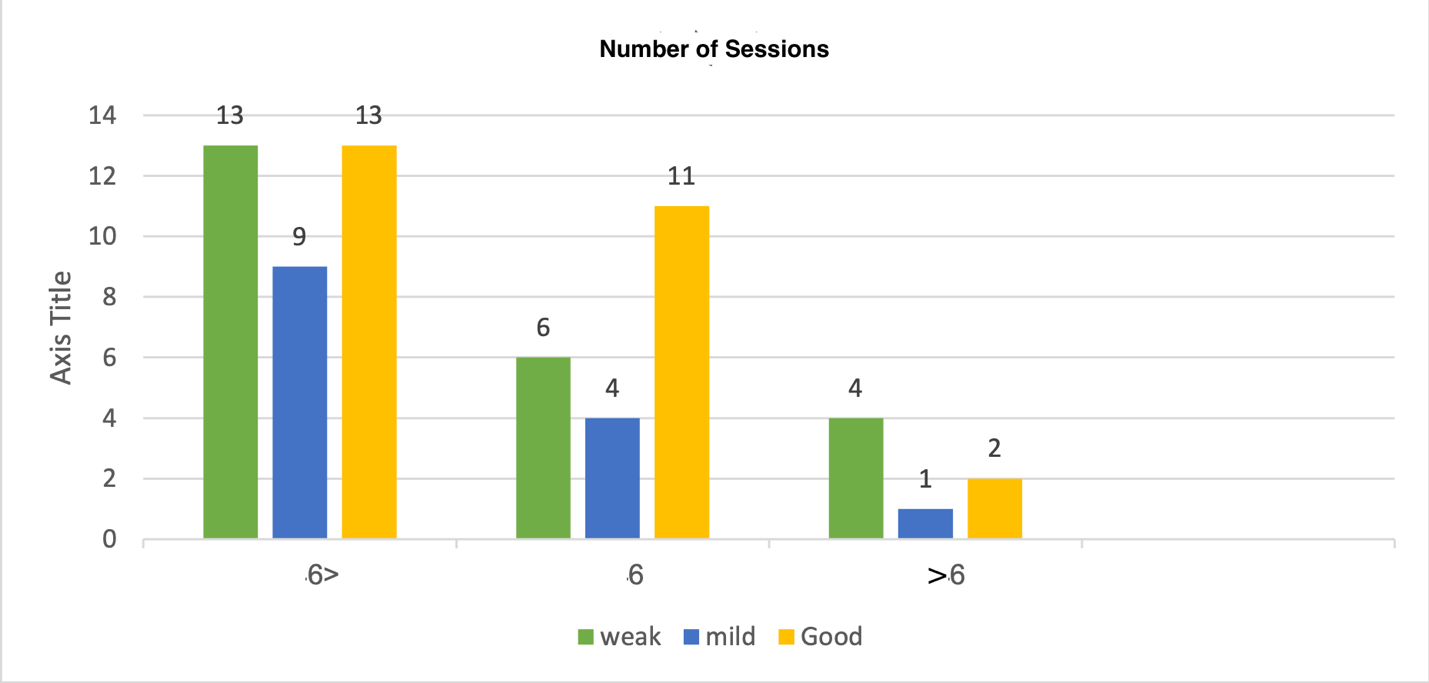


Figure 8': Frequency of Blind Dermatologist Assessments Based on the Number of Mesotherapy Sessions.

The satisfaction of the blind physician based on the number of mesotherapy sessions was examined using the chi-square test and was found to be statistically non-significant (p-value = 0.636). The satisfaction of the blind dermatologist based on the type of mesotherapy group, considering the variable of the number of mesotherapy sessions, was analyzed using the Chi-square test, which revealed no statistically significant difference (p-value: 0.209) (Table 3’) (Figure 9’).

Table 3': Blind Dermatologist Assessment Scores in Different Mesotherapy Groups Based on the Number of Sessions. A p-value < 0.05 is considered significant.

*Figure 9’: Blind Dermatologist Assessment Scores in Different Mesotherapy Groups Based on the Number of Sessions.*

1. **Pattern Loss:**

Figure 10’ demonstrates the distribution of female- and male- pattern loss in the study.


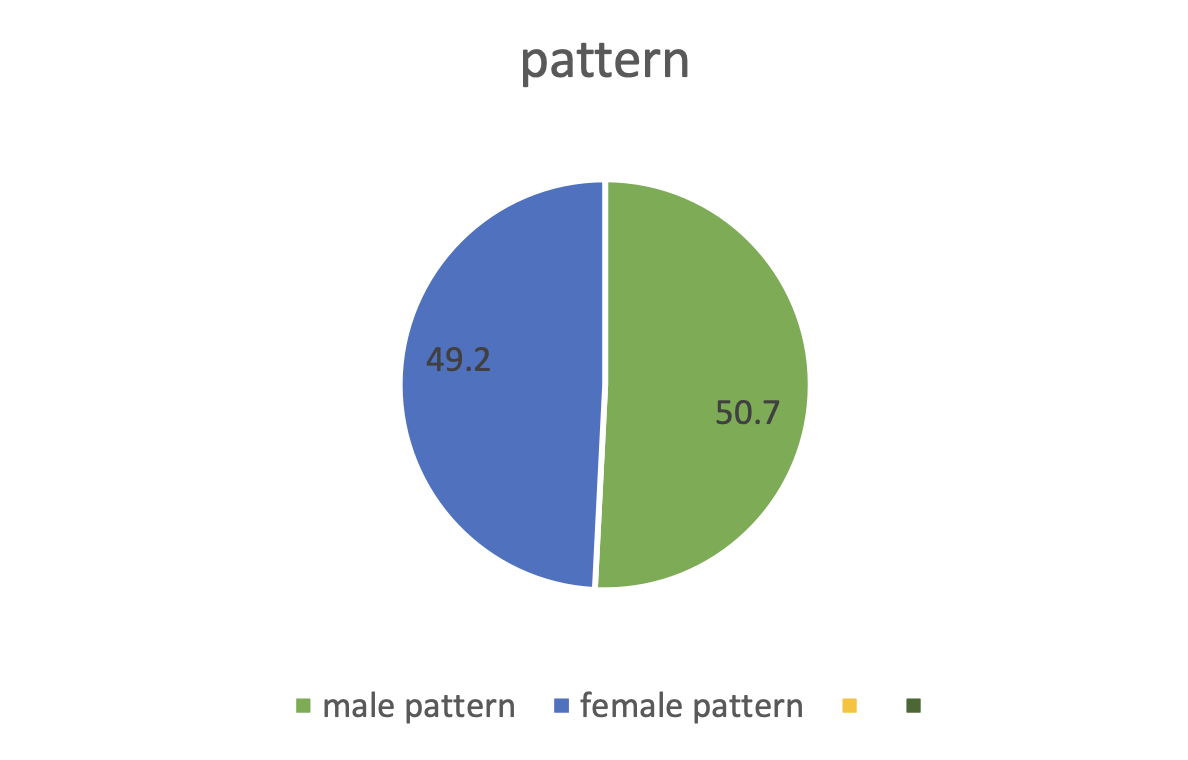


*Figure 10’: Distribution of Female and Male Pattern Hair Loss in the Current Study.*

In comparing the two groups, we found that 45.2% of individuals in the female pattern group achieved the highest level of satisfaction, while 37.6% of individuals in the male pattern group did. Therefore, patients with the female pattern exhibited higher satisfaction than those with the male pattern (Figure 11’).
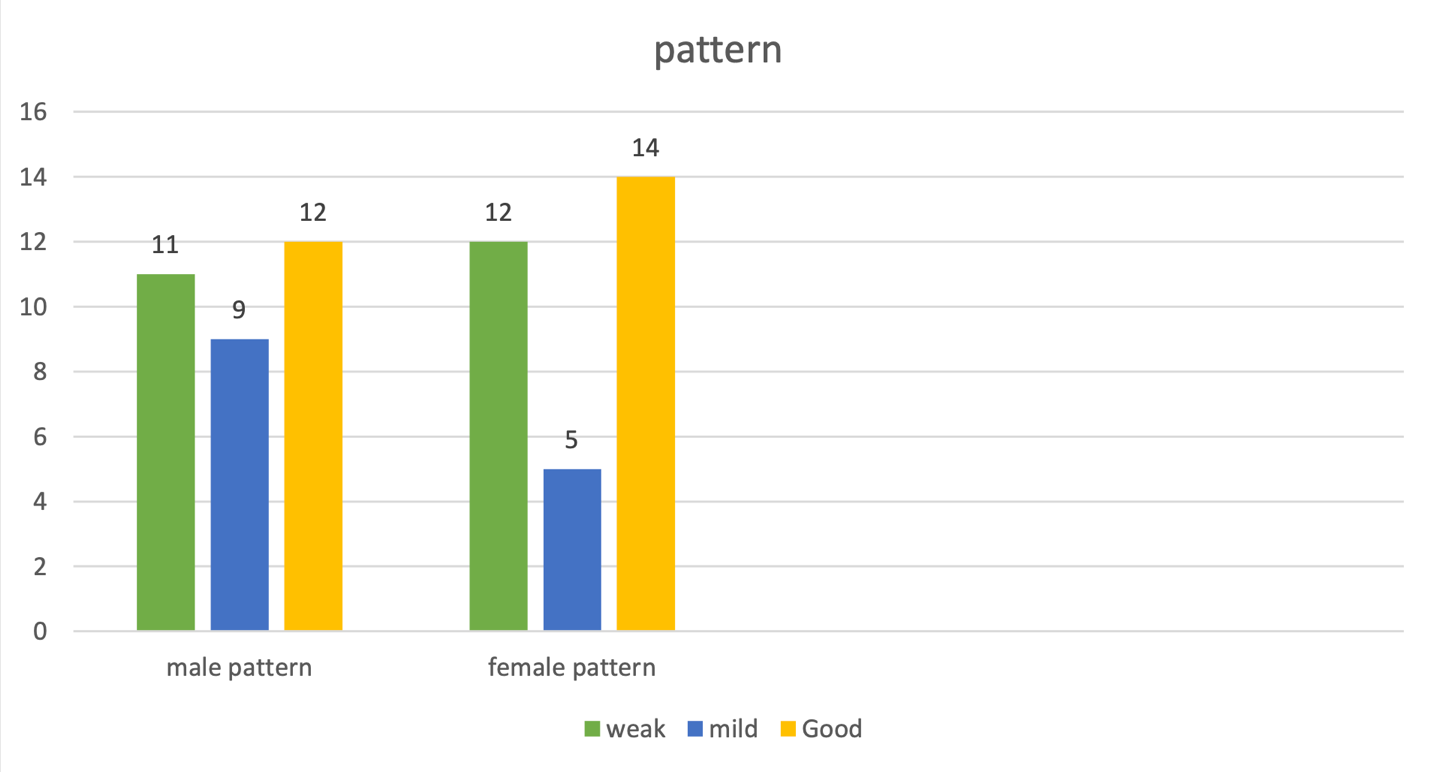


*Figure 11’: Blind Dermatologist Assessment Scores Based on Hair Loss Pattern.*

The satisfaction of the blind physician based on the alopecia pattern was examined using the Chi-square test, which revealed no statistically significant difference (p-value: 0.516). The satisfaction of the blind physician based on the type of mesotherapy used, considering the variable of hair loss pattern (p-value: 0.046), was analyzed and found to be statistically significant. (p-value=0.046) (Figure 12’) (Table 4’).

Table 4': Blind Dermatologist Assessment Scores in Different Groups Based on Hair Loss Pattern.

Figure 12': Blind Dermatologist Assessment Scores in Different Groups Based on Hair Loss Pattern.

1. **Grade of AGA:**

The frequency of grade of AGA is shown in Figure 13’.


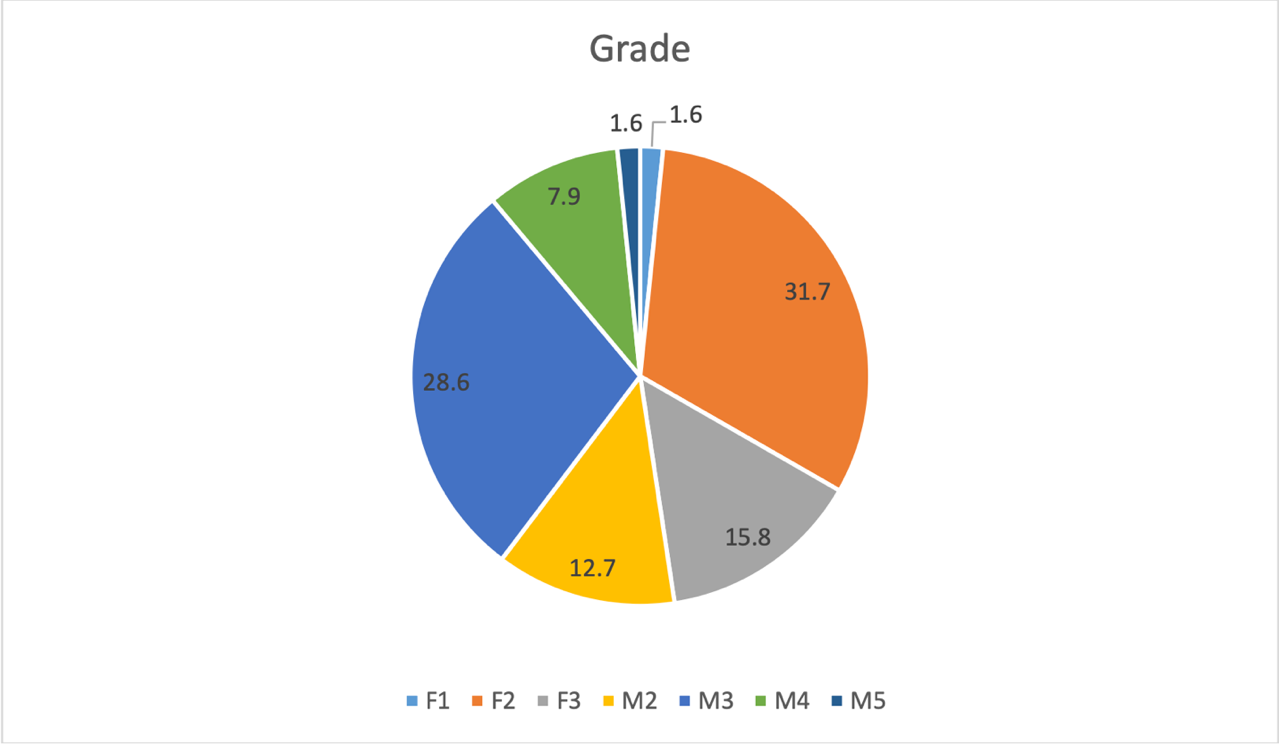


Figure 13': Frequency of Androgenetic Alopecia (AGA) Grades in the Current Study.

In this study, the satisfaction of the physician based on the grade of androgenetic alopecia at the start of treatment was examined using the Chi-square test, which revealed no statistically significant difference (p-value: 0.764) (Figure 14’).


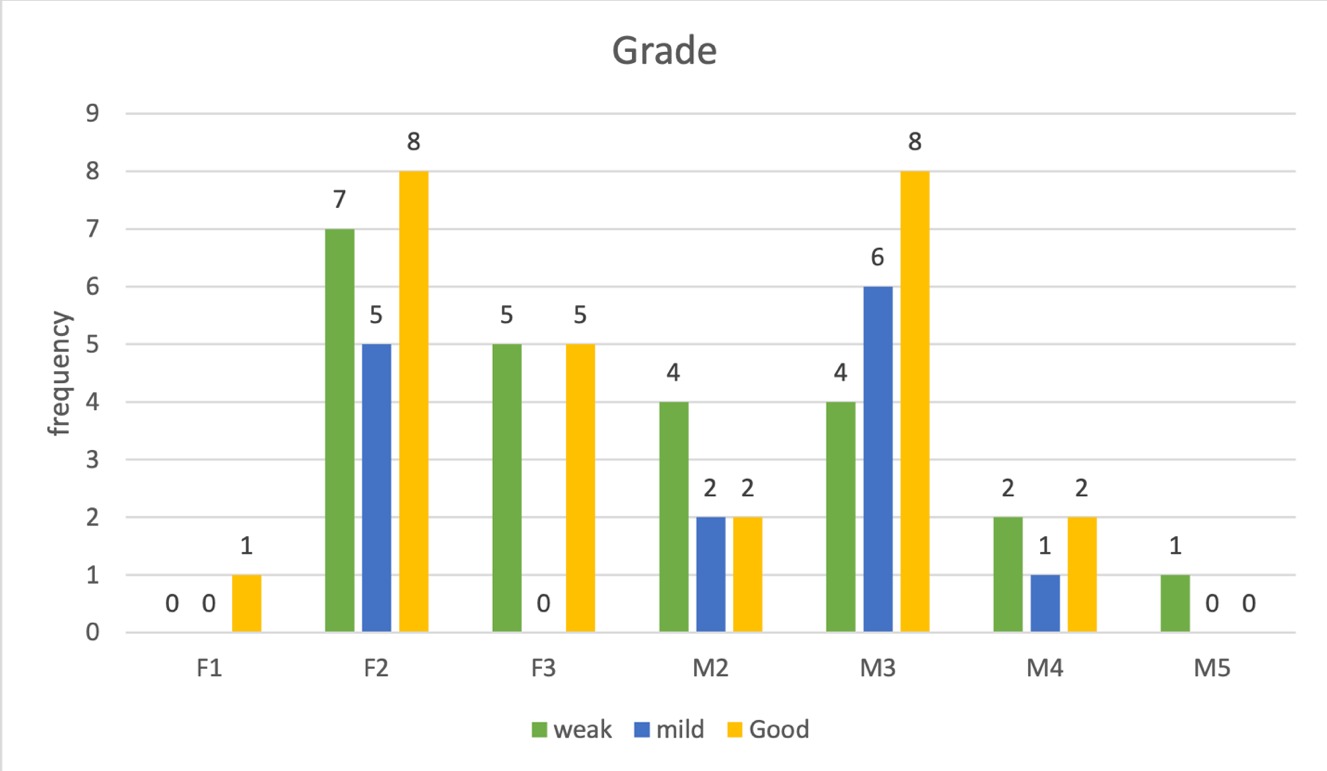


Figure 14': Frequency of Blind Dermatologist Assessments Based on AGA Grades.

The satisfaction of the blind physician based on the type of mesotherapy drug, considering the variable of hair loss grade, was analyzed using the Chi-square test, which revealed no statistically significant difference (p-value: 0.239) (Table 5’)(Figure 15’).

Table 5': Blind Dermatologist Assessment Scores in Different Groups Based on AGA Grades. A p-value < 0.05 is considered significant.

Figure 15': Figure 15: Blind Dermatologist Assessment Scores in Different Groups Based on AGA Grades.

1. **Past Medical History (PMH):**

Figure 16’ demonstrates the frequency of medical history in the participants.


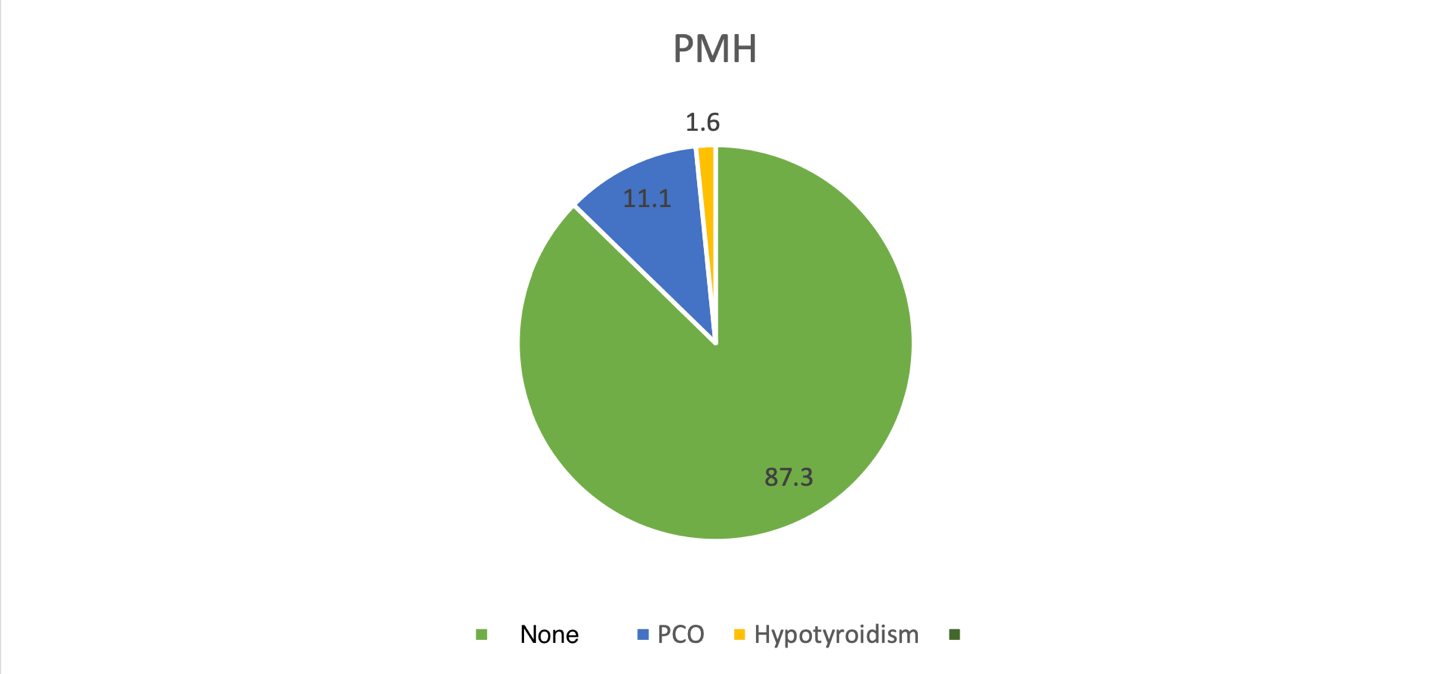


Figure 16': Frequency of PCOD and Hypothyroidism in the Current Study.

In this study, the satisfaction of the dermatologist based on the presence of underlying hormonal conditions was examined using the Chi-square test, which revealed no statistically significant difference (p-value:0.125) (Figure 17’).


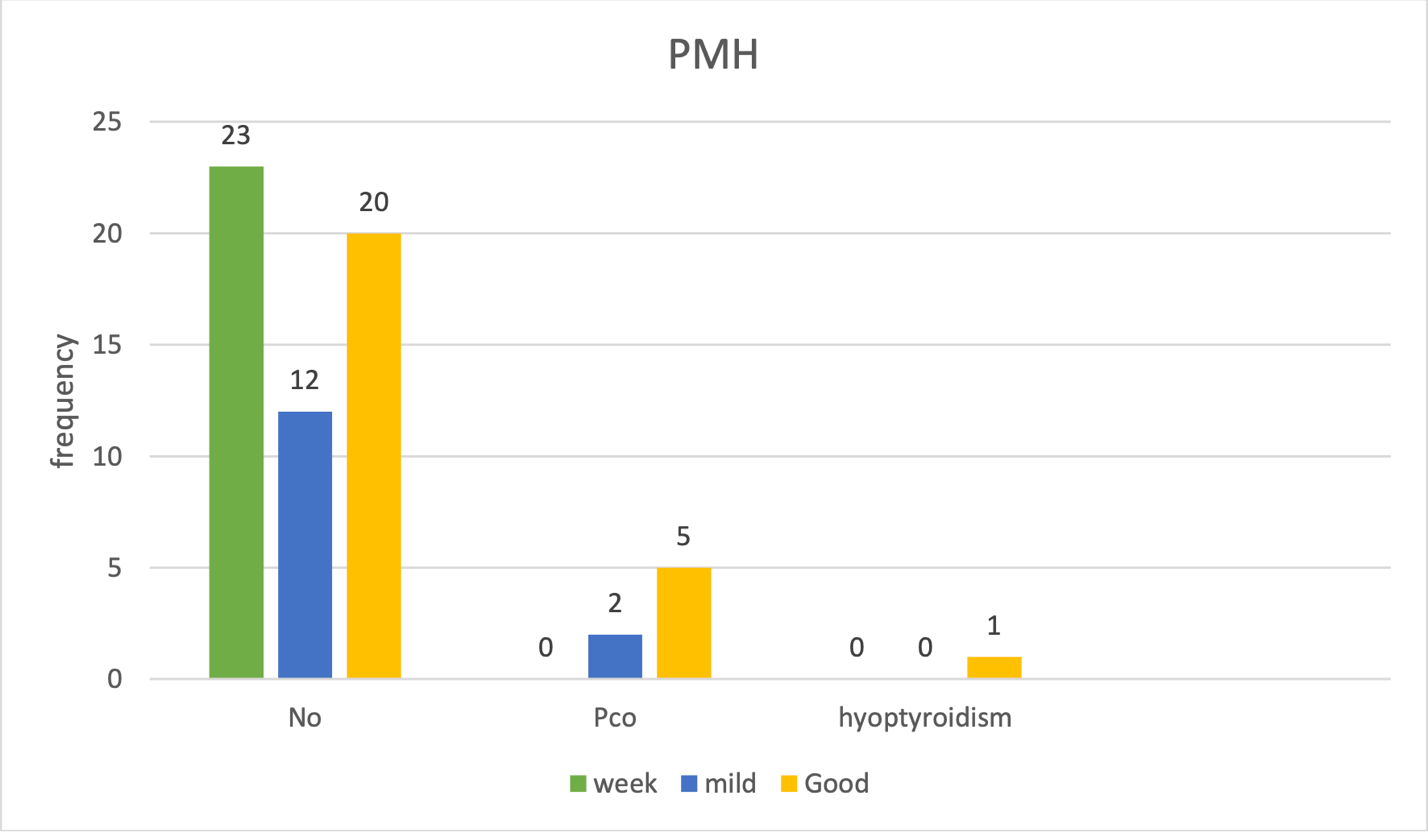


Figure 17': Blind Dermatologist Assessment Scores Based on PMH.

The satisfaction of the blind dermatologist based on the type of mesotherapy drug, considering the variable of past medical history (PMH), was analyzed using the Chi-square test, which revealed no statistically significant difference (p-value: 0.434) (Table 6’) (Figure 18’).

Table 6': Blind Dermatologist Assessment Scores in Different Mesotherapy Groups Based on Past Medical History (PMH). A p-value < 0.05 is considered significant.

Figure 18': Blind Dermatologist Assessment Scores in Different Mesotherapy Groups Based on Past Medical History (PMH).

1. **Oral Medication:**

Figure 19’ demonstrates the frequency of oral medication in the participants.


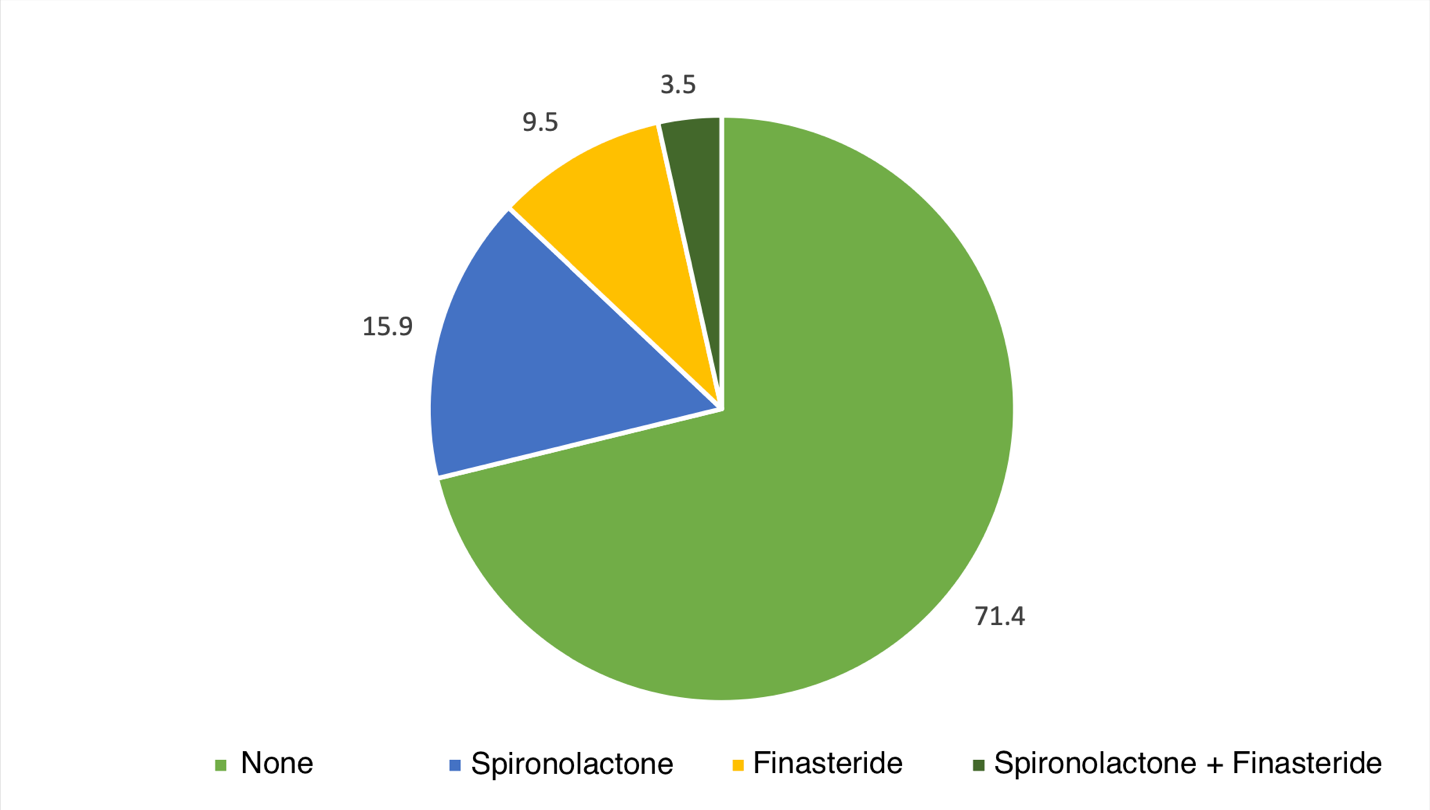


Figure 19': Frequency of Oral Medication in the Current Study.

In this study, the physician's satisfaction based on the oral medication used during treatment was examined using the Chi-square test, which revealed no statistically significant difference (p-value: 0.715) (Figure 20’).


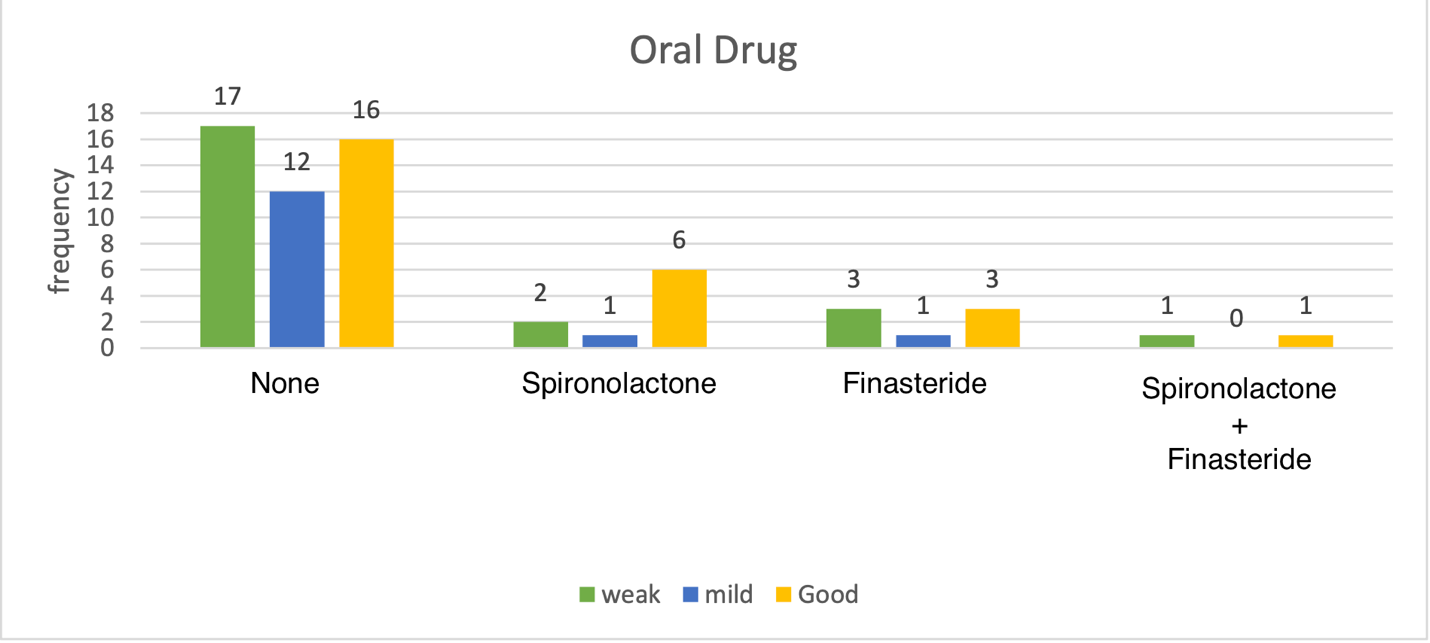


Figure 20': Blind Dermatologist Assessment Scores Based on Oral Medication.

In the current study, the satisfaction of the blind physician based on the oral medication used during treatment was examined using the Chi-square test, which revealed no statistically significant difference. (p-value: 0.362) (Table 7) (Figure 21’).

Table 7': Blind Dermatologist Assessment Scores in Different Mesotherapy Groups Based on Oral Medication. A p-value < 0.05 is considered significant.

Figure 21': Blind Dermatologist Assessment Scores in Different Mesotherapy Groups Based on Oral Medication.
